# Cellular and Molecular Comparison of Glioblastoma Multiform Cell Lines

**DOI:** 10.7759/cureus.16043

**Published:** 2021-06-29

**Authors:** Turan Demircan, Mervenur Yavuz, Egemen Kaya, Sıddıka Akgül, Ebru Altuntaş

**Affiliations:** 1 Genetics, Muğla Sıtkı Koçman University, Muğla, TUR; 2 Institute of Health Sciences, Muğla Sıtkı Koçman University, Muğla, TUR; 3 Surgery, Mugla Sitki Kocman University, Muğla, TUR; 4 Institute of Health Sciences, Aydın Adnan Menderes University, Aydın, TUR; 5 Institute of Natural Sciences, Muğla Sıtkı Koçman University, Muğla, TUR

**Keywords:** gbm, cell line, cellular and molecular biology, cancer cell biology, rt-qpcr

## Abstract

Glioblastoma multiform (GBM) is one of the most severe tumor types. It is highly invasive and characterized as a grade IV neoplastic cancer. Its resistance to chemotherapy-temozolomide (TMZ treatment)-in combination with tumor treating fields (TTFields), limits the cure of GBM. Therefore researchers are searching for new treatment options to increase the length of recurrence time and improve overall survival for GBM patients. Several cell lines have been established and are in use to understand the molecular basis of GBM and to test the developed drugs. On one hand, it is highly advantageous to utilize multiple cell lines with different genetic backgrounds to gain more insight into the characterization and treatment of the disease. However, on the other hand, characteristics of these cell lines such as proliferation rate, invasion, and colony formation capacity differ greatly among these cells. Hence, a detailed comparison concerning molecular and cellular features of commonly used cell lines is essential. In this study, cell proliferation and apoptosis rate, cell migration capacity, and gene expression profile of U87, Ln229, and SvGp12 cells have been investigated and compared.

## Introduction

Glioblastoma multiforme (GBM) is the most aggressive and malignant brain tumor [1]. Due to its complex nature, despite the efforts in decades of research, much about the molecular and cellular basis of GBM is still unknown. Rapid and infiltrative growth, invasion, and migration potential are the main characteristics of GBM. Furthermore, central necrosis and microvascular proliferation features of GBM might be additional contributing factors to the aggressiveness of cancer. Surgery or radiation therapy is not very effective to treat the GBM considering its high capability of invasion and infiltration [2]. Difficulties in the detection of tumor cells and migration of these cells to the contralateral hemisphere are the main limiting factors for surgical resection [3]. Infiltration into healthy tissues helps the GBM cells to escape from chemo and radiation therapy. Therefore, the utilization of available models such as GBM cell lines is essential to understand the heterogeneity of GBM and to enhance the understanding of glioma biology.

Results obtained from GBM in-vitro studies are less translatable to humans, however, they are still valuable enough to provide a basis for in-vivo researches. The major drawback to using 2-D models is its restricted capacity to simulate the in-vivo conditions. On the other hand, in-vitro models are good complementary options to in-vivo animal models regarding the shortcomings of animal studies such as being time-consuming, ethically problematic, expensive, and complex. Moreover, the easiness of manipulations in cell cultures and getting a fast response to environmental modulation are the other main advantages of using cell lines. Hence, using several GBM cell lines with different genetic backgrounds is of great importance to expanding our current knowledge on GBM [4]. Molecular signature specific to cell lines alters the cells' cellular capacity in proliferation, migration, and metastasis [5]. The objective of this article was to compare the cellular and molecular capabilities of U87 and LN229 GBM, and healthy SVGp12 cells.

## Materials and methods

Cell culture maintenance

Human GBM cells (LN229 and U87) and human astroglia cells (SVGp12) were provided from our stock (American Type Culture Collection [ATCC] catalog numbers: CRL-2611™, HTB-14, CRL-8621; respectively). U87 and SVGp12 cells were cultured in high-glucose Dulbecco’s modified eagle medium (DMEM) (cat. no. D6429; Sigma-Aldrich, St. Louis, MO) supplemented with Gibco 1% penicillin/streptomycin (pen/strep) (Thermo Fisher Scientific, Waltham, MA) and Gibco 10% heat-inactivated fetal bovine serum (FBS) (Thermo Fisher Scientific, Waltham, MA). For LN229 cells, DMEM supplemented with 5% FBS was used. All cell lines were maintained in a humidified chamber containing 5% CO2 95% air at 37°C. The medium was replaced three to four times per week. Cells were passaged at 80% confluency throughout the experiment. For the following assays, all cell lines were harvested with Gibco 0.25% Trypsin-EDTA (Thermo Fisher Scientific) and counted with a Thoma cell counting chamber (ISOLAB, Eschau, Germany) using 0.4% Trypan Blue Solution (Thermo Fisher Scientific). For all following assays, data have been obtained from three independent experiments with three replicates.

MTT assay

To elucidate the proliferation rate of the investigated cell lines, an MTT assay was used. For each cell line, 0.1x10^5^ cells/well were seeded to a 96-well plate (Thermo Fisher Scientific, Waltham, MA) in 100 μL culture media for 24 hours for attachment of cells. Then, the culture medium was replaced, and cells were incubated for another 24 hours in the freshly replaced medium. Following the incubation, MTT assay was performed using CellTiter 96® Non-Radioactive Cell Proliferation Assay kit (cat. no. G400; Promega, Madison, WI) according to the manufacturer’s protocol. Briefly, 20 μL dye solution, a filtered solution of tetrazolium dye (MTT), provided within the kit (cat. no. PartG402A; Promega) was added to each well, and cells were further incubated for four hours. Then, 100 μL solubilization solution/stop mix (cat. no. PartG401A; Promega) contained in the kit was added into wells to stop cells to form further formazan crystals. Afterward the overnight incubation, formazan crystals were detected with SpectraMax®i3 (Molecular Devices, San Jose, CA) and absorbances were recorded at 570/750 nm.

Colony formation assay (CFA)

Cells were cultured in a 96-well plate (ThermoFisher Scientific) with 0.02x10^5^ seeding density in 100 μL DMEM and incubated in the aforementioned culturing conditions. Following the medium change after 24 hours, the cell media was replaced every other day for five days. After cells reached a minimum of 80% confluency, the cells were fixed with 150 μL 100% methanol (cat. no. 1.06009.2511, Merck Group, Darmstadt, Germany) throughout 20 mins incubation at room temperature and then formed colonies were stained with 100 μL 0.2% Crystal Violet Solution (cat. no. C077; Sigma-Aldrich) for 15 mins at room temperature. To eliminate the background staining the cells were washed twice with 100 μL ddH2O. The plate was left to dry out overnight. Colonies were photographed then counted with the ImageJ program “ColonyCounter” plugin. 

Wound-healing assay

0.5x105 cells/well were seeded to a 24-well plate (ThermoFisher Scientific) in 1 mL culture media and incubated for 24 hours. Upon incubation, the culture medium was replaced, and cells were scratched straightly using 200 μl pipette tips. Finally, the cells were photographed at 0th-h, 6th-h, and 24th-h to compare the migration capacity of cell lines. ImageJ 1.52v image analysis software (htpp://imagej.nih.gov/ij) was employed to analyze and quantify the wound closure rates. 

qRT-PCR assay

The expression of PCNA, TP53, DNMT1, ATM, CCNE1, BRCA1, BRCA2, and MKI67 genes at the mRNA level was compared among cell lines by the RT-qPCR method described elsewhere [6,7]. U87, LN229, and SVGp12 cells were seeded with 3x10^5^ density on a 6-well plate (ThermoFisher Scientific, USA) in a 1 mL medium with 3 replicate, incubated for 24 hours, and then the culture mediums were replaced. Following the next 24th-h incubation, cells were harvested using Gibco 0.25% Trypsin-EDTA and washed with 1 mL PBS (cat. no. 003002; Thermo Fisher Scientific). The total RNA of the cells was isolated using Trizol reagent according to the manufacturer’s instructions. The quantity and quality of isolated RNA samples were determined by Qubit assay and 1% agarose gel, respectively. To perform cDNA synthesis, the SensiFAST cDNA synthesis kit (cat. no. BIO-65053; Meridian Biosciences Inc., Cincinnati, OH) was used by following the manufacturer’s protocol. Gene expression analyses were conducted using gene-specific primers and the SensiFAST SYBR No-ROX kit (cat. no. BIO-98005; Meridian Biosciences Inc.) according to the manufacturer’s instructions.

Statistics

Statistical analyses were performed using GraphPad Prism 5.0 (GraphPad Software, Inc., San Diego, CA). The normal distribution of the data was tested by using the Kolmogorov-Smirnov method. One-way analysis of variance (ANOVA) followed by Tukey’s test as a post hoc test was employed to analyze the statistical differences among groups. The results of statistics were presented as mean ± standard error of the mean (SEM). P-value lower than 0.05 was accepted as statistically significant (*p < 0.05, **p < 0.01, ***p < 0.001).

## Results

MTT assay revealed the cell division differences among the cell lines

**Figure 1:**
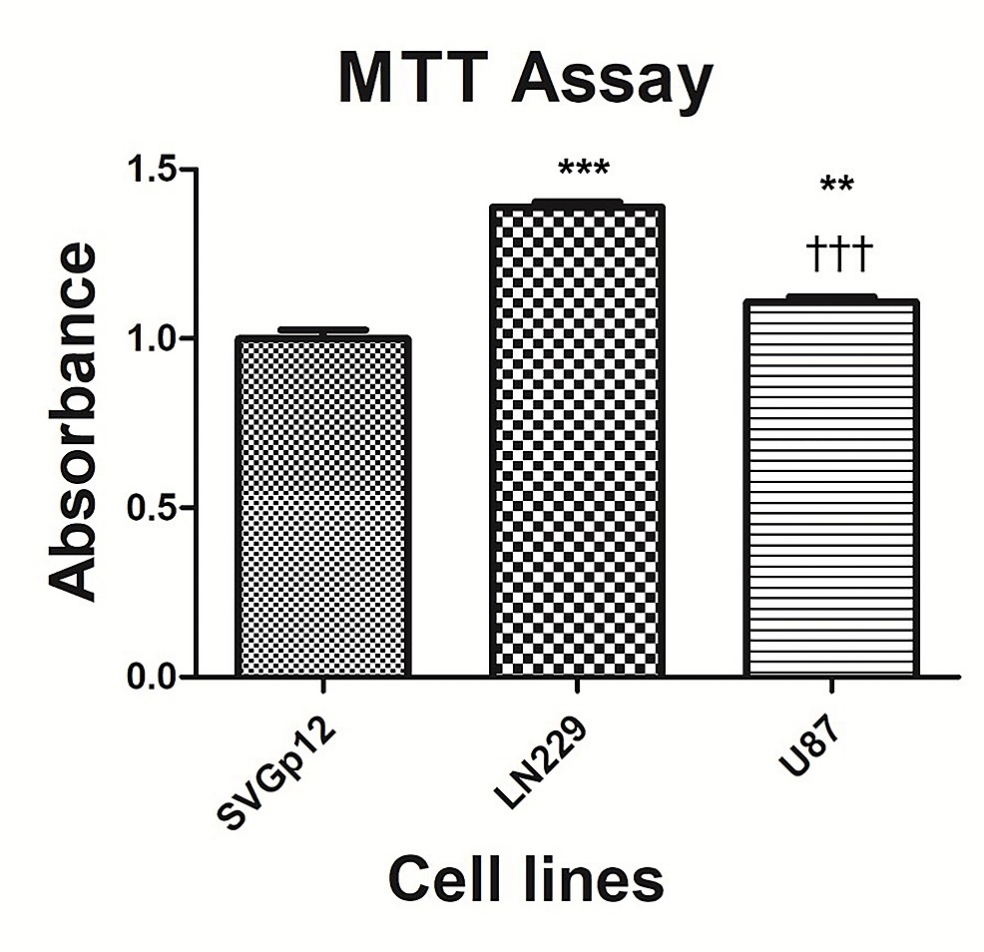
MTT Assay Comparison of the cell viability of SVGp12, LN229, and U87 cells. The cells were cultured under optimal conditions for 24 hours. Cell viability was evaluated by MTT cell viability assay. Data are the average (mean) results of three independent experiments. Star U87; LN229 vs Control; Plus U87 vs LN229. (**p < 0.01, ***p < 0.001).

The proliferation rate of cell lines was measured by MTT assay (Figure 1). Statistical analysis of obtained data indicated that LN229 cells which had the top proliferation rate, were found to be statistically different (p<0.001) from the SVGp12 cells, and U87 cells were significantly more proliferative than the SVGp12 cells (p<0.001). Notably, a significant difference between LN229 and U87 cells was observed (p<0.01).

CFA confirmed the MTT assay result

**Figure 2:**
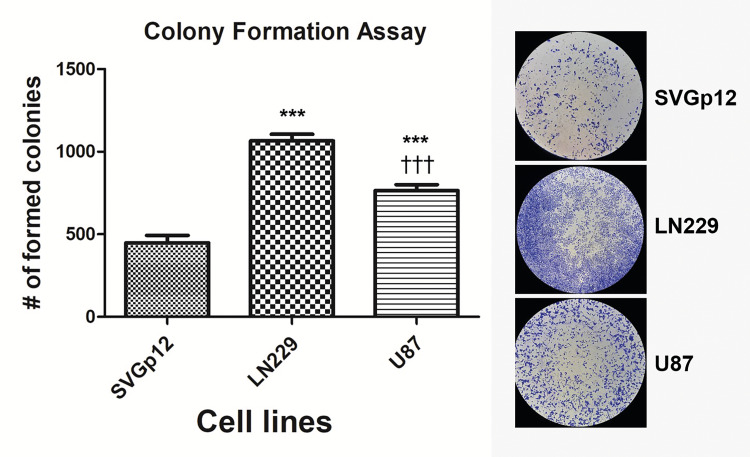
Colony formation assay Colony formation assay was used to compare the colony-forming ability of SVGp12, LN229, and U87 cells. Representative images of cell lines and the calculated numbers of formed colonies are displayed. Data are from three independent experiments. Star U87; LN229 vs Control; Plus U87 vs LN229 (***p< 0.001).

Colony formation assay was performed to extend the results of the MTT assay. Regarding the number of the formed colonies among the cell lines, LN229 cells demonstrated the highest colony-forming capacity followed by U87 and SVGp12 cells (Figure 2). Statistical analysis based on pairwise comparisons highlighted the significant difference in the colony-forming ability for each compared pair (p<0.001). 

Migration capacity differs considerably among cell lines

**Figure 3:**
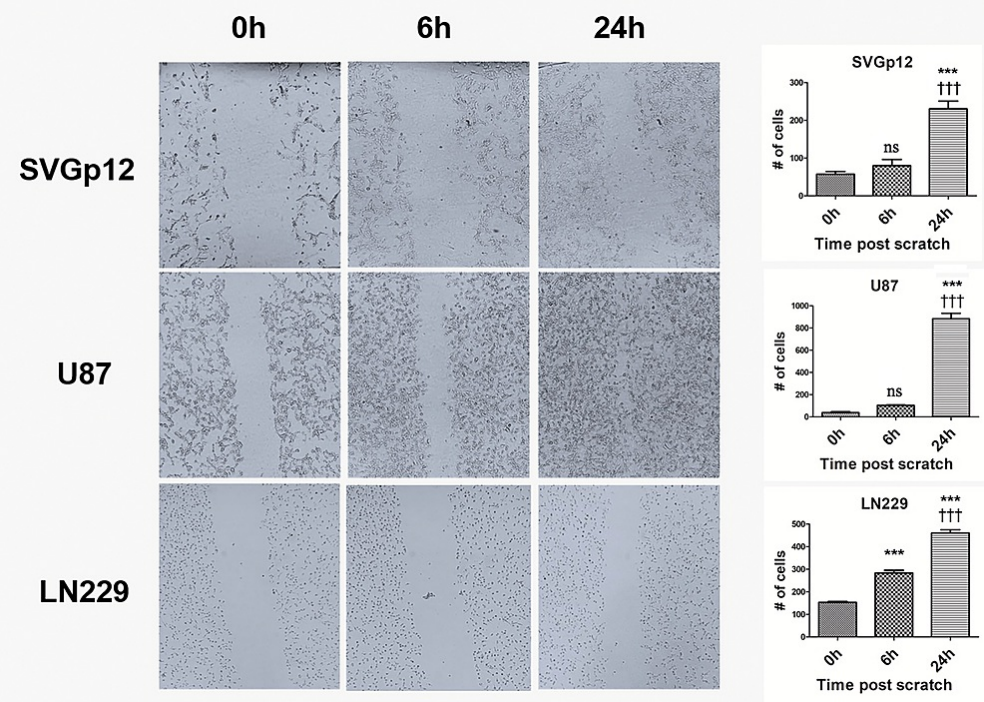
Wound healing assay Representative wound healing images of SVGp12, LN229, and U87 cells at 0, 16, 24 h. The number of migrated cells to the wound area was quantified using ImageJ. The average (mean) results of three independent experiments were presented. Star U87; LN229 vs Control; Plus U87 vs LN229 (*** p < 0.001).

To evaluate the migration capacity of investigated cell lines, a wound healing assay was employed. A variation in wound closure success was noted among the cell lines (Figure 3). The scratch healing rate for U87 cells was remarkably higher than LN229 and SVG p12 cells which displayed only partial healing (Figure 3). Compared to 0th-h, the number of migrated cells to the wound area was found statistically significant only for the 24th-h time point of both U87 and SVGp12 cells (p<0.001). On the other hand, for LN229 cells both 6th and 24th-time points were significantly close to the wound area in comparison to 0th-h (p<0.001).

Expression levels of cell cycle and DNA repair genes in cell lines

**Figure 4:**
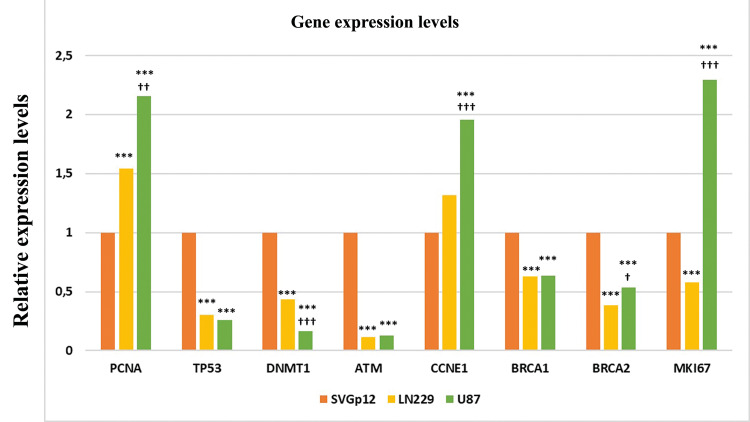
Gene expression analysis Gene expression of selected genes in SVGp12, LN229, and U87 cells was determined by using quantitative real-time PCR (RT-qPCR). Each cell line was assayed three times and each assay included three replicates. Star U87; LN229 vs Control; Plus U87 vs LN229 (*p < 0.05, **p < 0.01, ***p < 0.001).

To check whether the molecular profile of cell lines was similar or not, quantification of the cell cycle, tumor suppressor, and DNA repair genes at the RNA level was conducted by using the qRT-PCR method. Glyceraldehyde 3-phosphate dehydrogenase (GAPDH) was used to normalize the data and relative expression levels of selected genes are shown in Figure 4. Marker genes of cell proliferation such as PCNA and MKI67 were found to be significantly upregulated in both LN229 and U87 cells (p<0.001). There was also a significant difference in expression levels of PCNA and MKI67 between LN229 and U87 cells (p<0.001). DNMT1, a DNA methyltransferase gene responsible for the maintenance of DNA methylation pattern, was significantly downregulated in LN229 and U87 cells compared to SVGp12 cells. Expression of tumor suppressor and DNA repair-related genes TP53, ATM, BRCA1, and BRCA2 significantly reduced in LN229 and U87 cells compared to SVGp12 cells. A considerable expression level difference of the BRCA2 gene between LN229 and U87 cells was noted.

## Discussion

GBM, an astrocyte-originated malignant cancer, is the most frequent type of central nervous system (CNS) cancer. Genetic and epigenetic alterations in GBM cause distinct mutations in diverse subgroups which eventually leads to variation in clinical outcomes [8]. The GBM cell lines used in this study, LN229 and U87, have various genetic alterations and possibly diverged mechanisms leading to tumor development. Type of mutations in cancer cells have a remarkable impact on their proliferation, migration, invasion, and metastasize capacity [9]. Therefore, it is important to consider these differences while using several GBM cell lines. Typical LN229 cell genetic background is characterized by the mutated TP53 gene, homozygous p16 and p14ARF deletion, and wild-type PTEN gene. By contrast with the LN229 cell, the U87 cell has a wild-type TP53 gene and a mutated PTEN gene with homozygous p16 and p14ARF deletion. 

In this study, first of all, the proliferation rate of the cell lines was evaluated with MTT and CFA assays. Experiments were set as starting with the same cell number for each cell line and upon the end of the experiment, cell number was measured by employing these methods. As a result of both assays, LN229 and U87 cells displayed a higher absorbance value and the number of formed colonies compared to SVGp12 cells. As another noteworthy observation, these values for LN229 cells were found significantly higher than U87 cells. In addition to higher proliferative and survival capacity, a lower apoptosis rate might be the possible contributor for getting a relatively higher number of cells. Further experiments to assess the proliferation and apoptosis rate (such as EdU labeling and annexin-V staining, respectively) would provide more insights to determine the main factors accounting for the observed differences in MTT and CFA results among the cell lines. 

Wound healing assay is a commonly used, simple, and standard method to study cell migration [10]. The generated gap on a cell monolayer is filled by the migrating cells moving towards the gap and the migratory response is quantified to find the migration capacity of the examined cells [11]. The rate of gap closure is linked to the speed of the motile cells which move to the gap zone in a coordinated fashion. Our result demonstrated that U87 cells have the highest migration ability among all cell lines and this U87 wound closure rate was followed by LN229 cells. Both SVGp12 and LN229 cells exhibited a significant wound closure at the 24th timepoint, however, these cells could not completely heal the scratch. Although LN229 cells showed the highest proliferation and/or survival capacity, the higher wound closure potential in U87 cells compared to LN229 cells indicates that the migration level of U87 cells was higher. This finding is aligned well with a previous study conducted on GBM cells [12].

As a complementary analysis to cellular experiments, changes at the transcriptional level of selected genes were investigated. Proliferating cell nuclear antigen (PCNA) gene plays an essential role in DNA replication as the processivity factor for DNA polymerase and therefore is commonly used as a cell division marker; MKI67, another proliferation-related gene, takes crucial roles in the formation of a ribonucleoprotein layer, the perichromosomal layer (PCL), to prevent the aggregation of mitotic chromosomes [13]. Expression levels of these cell-cycle regulating genes were significantly higher in LN229 and U87 cells compared to SVGp12 cells. Considering the favorable gene expression program of cancer cells in the proliferation process, higher expression of cell-cycle related genes in GBM cell lines in comparison to SVGp12 cells was expectable and validated by our data. DNA repair and tumor suppressor related genes such as ataxia-telangiectasia mutated (ATM) gene, breast cancer type I and type II susceptibility genes (BRCA1 and BRCA2, respectively), and TP53 were found significantly lower in LN229 and U87 cells than SVGp12 cell line, suggesting that negative regulators of cell proliferation are suppressed in these cells. DNA methyltransferase I (DNMT1) [14], an enzyme that maintains the methylation pattern of replicated DNA was found to be significantly lower in both U87 and LN229 cells. Considering the accumulated literature on the decreased global methylation in cancer cells due to the reduced gene expression level of DNMT1, the lower expression of DNMT1 in GBM cell lines is in harmony with previously published data. CCNE1, the G1/S-specific cyclin-E1 protein gene is required for G1/S transition and is an important cell-cycle regulator gene that positively regulates the cell cycle [15]. Statistical analysis revealed that the CCNE1 gene expression level was found significantly higher in U87 cells compared to LN229 and SVGp12 cell lines. Previous studies linked the higher CCNE1 mRNA levels in the U87 cell line with temozolomide (TMZ) resistance and the high expression of CCNE1 in U87 cells was confirmed by our data. TMZ is an important chemotherapy agent [16] acclaimed as the gold standard for the treatment of GBM and in a previous report, it was shown that glioma cells considerably became more sensitive to temozolomide treatment after BRCA2 knockdown [17]. Notably, BRCA2 levels in LN229 cells were significantly reduced in comparison to U87 cells. The significant difference in CCNE1 and BRCA2 mRNA levels between U87 and LN229 cells may lead to being sensitive or resistant to TMZ treatment. Therefore, molecular interrogation of heterogeneous GBM cells isolated from the patients might be guiding to enhance the effect of TMZ treatment by modulating the expression levels of CCNE1 and BRCA2 genes.

Here, we aimed to unveil the proliferative and migrative capacity of GBM cell lines, U87 and LN229, and make a comparison with healthy astrocyte cell line, SVGp12. Performed assays at the cellular level highlighted the higher proliferation and migration ability of GBM cells compared to SVGp12 cells and the molecular basis for this notion is examined. The results obtained from qRT-PCR correlated with CFA and MTT data. Overall, we hope that our results would contribute to understanding the cellular and molecular behaviors of GBM cell lines.

## Conclusions

In this study, we compared the cellular and molecular features of cell lines widely used in GBM studies. Detected differences among cell lines in tumorigenicity could provide new insights into understanding the obtained variation associated with the utilized cell line. Further studies are needed to explore the mechanistic link between the genetic background of cell lines and the observed phenotype.
